# Embryotoxic Effects of Pesticides in Zebrafish (*Danio rerio*): Diflubenzuron, Pyriproxyfen, and Its Mixtures

**DOI:** 10.3390/toxics12020160

**Published:** 2024-02-18

**Authors:** Júlia Robert de Sousa Teixeira, Augusto Monteiro de Souza, João Vitor de Macedo-Sampaio, Fabiano Peres Menezes, Bruno Fiorelini Pereira, Silvia Regina Batistuzzo de Medeiros, Ana Carolina Luchiari

**Affiliations:** 1 Department of Physiology and Behavior, Federal University of Rio Grande do Norte, Natal 59072-970, RN, Brazil; juliarobert.teixeira@gmail.com (J.R.d.S.T.); jvms581@gmail.com (J.V.d.M.-S.); 2 Graduate Program in Psychobiology, Biosciences Center, Federal University of Rio Grande do Norte, Natal 59072-970, RN, Brazil; 3 Department of Cell Biology and Genetics, Biosciences Center, Federal University of Rio Grande do Norte, Natal 59072-970, RN, Brazil; augustomonteiro25@gmail.com (A.M.d.S.); sbatistu@gmail.com (S.R.B.d.M.); 4 Brazilian Institute of Environment and Renewable Natural Resources (IBAMA), Rio Grande 96200-180, RS, Brazil; fabiano.menezes@ibama.gov.br; 5 Department of Biology, Federal University of São Paulo (UNIFESP), Diadema 09913-030, SP, Brazil; bf.pereira@unifesp.br

**Keywords:** agrochemical, mortality, teratogenicity, behavior, neurotoxicity

## Abstract

Diflubenzuron (DFB) and pyriproxyfen (PPF) are larvicides used in crops to control insect plagues. However, these pesticides are known to impact non-target organisms like fish and mammals. Here, we aimed at assessing the embryotoxicity of purified DFB, PPF, and their mixtures in a non-target organism—zebrafish. Zebrafish embryos were exposed to different concentrations for 120 h: 0.025, 0.125, 0.25, 1.25, 2.5, and 10 mg/L of purified PPF and purified DFB, while we used 0.025 mg/L PPF + 10 mg/L DFB (Mix A), 0.125 mg/L PPF + 10 mg/L DFB (Mix B), and 0.25 mg/L PPF + 10 mg/L DFB (Mix C) for the mixtures of PPF + DFB. We observed mortality, teratogenicity, and cardiotoxicity. For the neurotoxicity tests and evaluation of reactive oxygen species (ROS) levels in the brain, embryos were exposed for 120 h to 0.379 and 0.754 mg/L of PPF and 0.025 and 0.125 mg/L of DFB. We established the LC_50_ for PPF as 3.79 mg/L, while the LC_50_ for DFB was not determinable. Survival and hatching were affected by PPF concentrations above 0.125 mg/L, DFB concentrations above 1.25 mg/L, and the lower pesticide mixtures. PPF exposure and mixtures induced different types of malformations, while a higher number of malformations were observed for the mixtures, suggesting a potentiating effect. Pesticides diminished avoidance responses and increased the levels of ROS across all concentrations, indicating neurotoxicity. Our findings underscore the detrimental impact of PPF and DFB exposure, spanning from biochemistry to morphology. There is a critical need to reconsider the global use of these pesticides and transition to more ecologically friendly forms of pest control, raising an alarm regarding repercussions on human and animal health and well-being.

## 1. Introduction

With the rapid growth of the population, the use of pesticides has significantly intensified since the 1960s, largely driven by the “green revolution”—a period of extensive agricultural modernization [1]. Pesticides are chemical substances primarily employed in the agricultural sector [2] to control pests and diseases that pose a threat to crops, ensuring their health and productivity [3]. According to the Food and Agriculture Organization of the United Nations [4], the presence of active pesticide ingredients used in global agriculture reached 2.7 million metric tons (Mt) in 2020. In this scenario, the United States emerged as the leading user of pesticides, followed by Brazil and China. The Phytosanitary Pesticide System (https://agrofit.agricultura.gov.br/ accessed on 20 January 2024) currently authorizes the use of approximately 2962 pesticides in Brazil, which are regulated by the pesticide law—Law No. 7802, dated 11 July 1989—[5] that classifies them based on action, purpose, and prevention of risks associated with exposure.

According to the Brazilian Health Regulatory Agency [6], research conducted in 26 states of Brazil through the Pesticide Residue Analysis Program in Food reveals that roughly one-third of the daily food consumed by Brazilians is contaminated with pesticides. Similarly, the World Health Organization [7] reported that 3 to 5 million people experience pesticide (or their residues) intoxication each year, highlighting the need to reevaluate safe concentrations for their usage. For instance, in 2016, studies conducted by the International Agency for Research on Cancer (IARC) [8] demonstrated that the pesticide *lindane* has carcinogenic effects and causes immunosuppression in humans due to exposure.

Among the most applied pesticides to crops, Diflubenzuron (DFB), a urea derivative, and Pyriproxyfen (PPF), a pyridine derivative, are larvicides that regulate insect growth by inhibiting chitin formation, the primary component of arthropods exoskeleton [9]. These substances find use in various crops such as cotton, rice, and soybeans (https://agrofit.agricultura.gov.br/ accessed on 20 January 2024). However, the effects of these pesticides are not restricted to the insects that feed on the crops. For example, Maduenho and Martinez [10] demonstrated a reduction in the number of red blood cells and hemoglobin content in the juvenile Curimbatá fish (*Prochilodus lineatus*), as well as hepatic alterations after acute exposure for 6, 24, and 96 h to DFB. More than that, 28-day oral exposure to PPF caused a reduction in body and organ weight in male Swiss albino mice [11]. Thus, the pesticides can cause effects in non-target organisms and continuous exposure to these chemicals should be carefully evaluated. Moreover, it is worth noting that crops are treated with multiple pesticides to combat various pests, including fungi, weeds, and insects. Consequently, agricultural production may accumulate diverse concentrations of different pesticides, which can be ingested and accumulate in non-target organisms. Continuous exposure to combinations of various pesticides occurs as a result of the consumption of crops by humans [12]. Although used in low concentrations, the long-term effects of these combined toxics on human and animals health are challenging to predict [13] and must be deeper investigated.

Therefore, this study aimed to evaluate the effects of DFB, PPF, and its mixtures in non-target organisms. Here we chose to use the zebrafish embryo and larvae to evaluate the pesticides effects on the development and behavioral response. Zebrafish is considered a promising model organism for assessing acute and chronic toxic effects [14] due to its 70% genetic similarity to the human genome, high reproducibility, and rapid development. Additionally, the transparency of zebrafish embryos allows for a comprehensive assessment of toxic effects on their development [15,16,17]. Hence, this study enables the understanding of the combined effects of two widely used pesticides present in the environment and in the human diet, consequences that cannot be estimated from their use in agricultural activities. While several studies have investigated the individual toxicity of DFB and PPF on various species, including fish, none have examined the combined effects of these pesticides to identify potential synergistic or antagonistic interactions. We hypothesize that exposure to both pesticides, even at low concentrations, may induce toxicological effects that surpass the impact of either pesticide alone.

## 2. Materials and Methods

### 2.1. Ethical Note

Wild-type AB strains were diagnosed in male and female zebrafish (*Danio rerio*), in accordance with the normative resolution CONCEA (National Council for the Control of Animal Experimentation) number 34. The maintenance procedures and experimental protocols were approved by the Ethics Committee for Animal Use from the Federal University of Rio Grande do Norte (CEUA, institutional certificate No. 329.006/2023). Animals’ health and well-being were monitored daily during all experimental phases. In all phases of this study, the authors complied with the ARRIVE guidelines.

### 2.2. Animals and Housing

Adult zebrafish between 4 and 6 months of age were housed at the FishLab (Federal University of Rio Grande do Norte—UFRN). The animals were fed twice a day with commercial flake food (Nutricon^®^ feed, Nutriflakes, Araçoiaba da Serra, Brazil) and *Artemia salina* nauplii (*Artemia salina* do RN^®^, Natal, Brazil) 24 h after hatching (live food). The fish were kept in an automated rack system (ZebTEC Active Blue Stand Alone—Tecniplast^®,^ Buguggiate, Italy) at a constant temperature of 28 °C, conductivity of 6μOsm, and pH of 7.2. The laboratory photoperiod was set at 14L:10D (Light:Dark), with lights on at 7:00 am (ZT0).

### 2.3. Obtaining Embryos

Adult zebrafish of the AB strain (6 months, 0.40 ± 0.03 g) from four breeding stocks were placed in breeding tanks (3 males: 2 females/tank), where males and females were physically separated (only visual and chemical contact) for 12 h (overnight). The partition separating animals was removed during the first hour of the following morning light cycle, and the fish were permitted to mate for 60 min. This procedure ensured knowledge of the exact time of the egg fertilization window. Then, the eggs from the different matrices were collected from each rearing tank, and were transferred together to Petri dishes, where they were observed under a magnifying glass to check fertilization and blastula formation at 3 h post-fertilization (hpf).

### 2.4. Acute Toxicity Test

Purified Diflubenzuron (DFB; Sigma-Aldrich Ref. 45446) and Pyriproxyfen (PPF; Sigma-Aldrich Ref. 37174) were dissolved in dimethyl sulfoxide at 1% (DMSO, Neon, Pa 1000 mL, Sigma-Aldrich, Barueri, Brazil) to produce the following concentrations for each product: 0.025 mg/L, 0.125 mg/L, 0.25 mg/L, 1.25 mg/L, 2.5 mg/L, and 10 mg/L. The doses used were based on the doses recommended by the WHO, which are 0.25 mg/L for diflubenzuron and 0.25–0.5 mg/L for pyriproxyfen. In view of the need to establish parameters such as LC_50_, we expanded the test range to higher and lower concentrations from that recommended, in respect to the solubility achieved in our preliminary tests. The concentrations of the mixtures of the two larvicides were: Mix A—0.025 mg/L PPF + 10 mg/L DFB, Mix B—0.125 mg/L PPF + 10 mg/L DFB, and Mix C—0.25 mg/L PPF + 10 mg/L DFB. Mixtures were selected based on the lowest concentration of each purified pesticide that caused toxic effects in the tests. Negative controls (water from the animal maintenance system and 1% DMSO) and a positive control (4 mg/L 3,4-dichloroaniline, Sigma-Aldrich) were included as control groups (OECD 236). For each concentration of the tested substances, 36 embryos were used and continuously exposed for 120 h in polystyrene well plates (24 wells), following OECD Guidelines No. 236 (Fish Embryo Acute Toxicity (FET) Test). The tests were conducted in triplicate, with 12 embryos per treatment concentration each time.

Every 12 h, the embryos were observed under a stereoscopic binocular microscope (80x magnification) to assess embryonic developmental stages, survival, and the occurrence of phenotypic abnormalities. Malformations of the head, tail, spinal cord, and the presence of pericardial edema, yolk sac edema, and changes in body pigmentation were evaluated. The distinction between normal and abnormal development was established by comparing the treated groups with the control group and using a zebrafish embryonic description [18].

To determine the LC_50_ (Lethal Concentration 50), which is a measure of the concentration of a substance that is lethal to 50% of a population, the data regarding the mortality rate at 120 h was used following OECD Guidelines No. 203 (Fish, Acute Toxicity Testing).

### 2.5. Cardiac Function

The cardiac function was observed at 96 hpf as indicated by the OECD Guidelines No. 236. To determine the heart rate, a count of 18 animals per group (control and treated groups) was conducted either live or via video, using a stereoscopic binocular microscope (80× magnification) that allows an enlarged view of the atrium and ventricle movements in the pericardium. The count was performed for 10 s, and thereafter, the beats were multiplied by six, resulting in a heart rate per minute for each individual [19]. Counting was performed in triplicate for each animal and the average value was used.

### 2.6. Behavioral Analysis

A batch of larvae exposed to pesticides for 120 h were subjected to optomotor and avoidance tests at 7 dpf (days post fertilization), following the methodology described by Creton [20] and Sousa et al. [21]. The concentrations used were based on the LC_50_ found for PPF (10% of the LC_50_: 0.379 mg/L and 20% of the LC_50_: 0.754 mg/L) and the lower concentrations of DFB(0.025 and 0.125 mg/L). Negative controls (water and 1% DMSO) were used. For both tests, PowerPoint projections were used on a computer screen, with a Petri dish (8.5 cm in diameter) containing the animals placed on the screen’s surface. Both behavioral tests were conducted in duplicates using 10 larvae each time. 

For the optomotor response test, 10 larvae from each treatment (control groups and pesticides’ groups) were separated into Petri dishes and exposed to a projection of the moving black and white stripes on a computer screen. The test was performed in duplicate. The movement pattern consisted of 1 min of stripes in one direction, followed by a 5 s interval with no stimulus (white screen), and then the stripes moved in the opposite direction for another 1 min. The optomotor response was recorded for 20 min. Every interval generated one picture (a total of 20 images extracted from each group) that was analyzed to extract the positions of each animal along the test. To analyze the larvae response, ImageJ (https://imagej.net/ij/index.html accessed on 20 January 2024) was employed to import the images and determine the orientation of the larvae in relation to the centroid. After identifying the X and Y axes, Microsoft Excel (https://www.microsoft.com/en-us/microsoft-365/excel accessed on 20 January 2024) was used to evaluate the accuracy of the larvae concerning the direction of the stripes and to indicate the positive (correct orientation toward the stripes movement) or negative (incorrect orientation) effect of the groups based on an average of each group. The total number of positive responses was compared between groups. The total number of animals (*n* = 20) and total number of images generated (20) were determined based on previous studies that support the robustness of the results [21,22].

For the avoidance test, a black circle projection with lateralized movement alternation was used. This test evaluated the behavioral pattern of the larvae concerning the distance from the moving black circle (10 cm in 3 s, which is equivalent to 3.3 cm/s). The aversive behavior of the larvae was evaluated during 10 min, during which images were registered every time the black circle was positioned at the upper left side of the plate (not visible to the larvae). A total of 10 images were obtained. Images were imported into ImageJ to obtain larval distribution compared to the centroid. Larvae were identified and the X and Y coordinates were exported to Microsoft Excel. Four quadrants of the plate were analyzed: upper right, upper left, bottom right, and bottom left. The closest quadrant to the stimulus (black circle) was the upper left area, which received value zero. Thus, larvae at the upper left quadrant scored zero in the analysis. The other quadrants received values related to their distance to the stimulus: upper right area scored 1, bottom left area scored 2, and bottom right (the farther area) scored 3. Thus, from the 10 larvae in each group (control groups and treated groups), each larvae scored a value related to its position. When the average of the group was closer to 3, it indicated that more larvae were in the bottom right quadrant that was farther from the aversive stimulus. On the contrary, when the average of the group was closer to 1, it indicated that a higher number of larvae was in the upper left quadrant that was closer to the aversive black circle.

### 2.7. Evaluation of Reactive Oxygen Species (ROS)

For the ROS analysis, the protocol described by da Silva Junior [23] was used. After 120 h exposure to different concentrations (PPF at concentrations of 0.379 and 0.758 mg/L, and DBF at concentrations of 0.025 and 0.125 mg/L), pools of 10 larvae per group were euthanized by cooling and washed three times with a chilled PBS solution at pH 7.4. The larvae were sonicated in 300 µL of PBS at 50% amplitude for 10 s and then centrifuged at 10,000× *g* for 10 min at 4 °C. The supernatant from the samples was collected, and a fluorescence probe, 2′,7′-dichlorodihydrofluorescein diacetate (H_2_DCFH-DA), was used to assess the presence of free radicals. A total of 40 µL of the homogenized larval solution was incubated with 40 µM H_2_DCFH-DA at 37 °C for 30 min in the dark. The measurements were performed in quintuplicate, in two analyses, using a multi-label microplate reader (GloMax^®^-Multi Detection System; Promega, Madison, WI, USA), with excitation at 490 nm and emission at 530 nm. It should be noted that fluorescence quantification was performed every 30 min (30, 60, 90, and 120 min). The ROS results were presented as a percentage (%) compared to the control group.

### 2.8. Statistical Analysis

The Log-rank test (Mantel–Cox) was used for the survival and hatching rate analysis. The LC_50_ was performed only for the percentage referring to PPF concentrations, as the pesticide DFB did not cause mortality. We used the ‘drm’ function of the drc package of the R program. This function allows to adjust different models of non-linear dose–response to binary data, using the maximum likelihood method. We specified the arguments fct = LL.2() and type = ‘binomial’ to indicate that we chose the two-parameter logistic function and that we have data of the binomial type. The two-parameter log-logistic function has the form:

$$f\left(x\right)=\frac{1}{1+\exp(b\left(\log\left(x\right)-\log\left(e\right)\right))}$$

where *x* is the dose, *f*(*x*) is the response, *e* is the effective dose of 50% (ED50) and *b* is the slope parameter. The LC_50_ was calculated as:

$$LC50=e.exp\left(\frac{1}{b}\right)$$

The LC_50_ was obtained from the ED function of the ‘drc’ package.

For the analysis of cardiac function, a one-way ANOVA was conducted to compare the mean heart rates across groups. The data related to embryo/larval developmental parameters and behavioral data were assessed for normality and homoscedasticity using the Shapiro–Wilk and Levene tests, respectively. To compare groups exposed to different concentrations of the pesticides, one-way ANOVA tests were conducted. Additionally, the two-way ANOVA test was performed to compare groups with different classifications of malformations (pericardium, sac, tail, spine, head, and eye). For the behavioral tests (optomotor response and avoidance test), one-way ANOVA was employed to compare the means of the groups (control groups and treated groups). To determine the ROS (Reactive Oxygen Species), a two-way ANOVA was conducted. The Tukey test was performed as a post hoc analysis when needed. All statistical analyses were conducted using Graphpad Prism (GraphPad Software Inc., San Diego, CA, USA) with a significance level of *p* < 0.05. 

## 3. Results

### 3.1. LC 50

**Figure 1:**
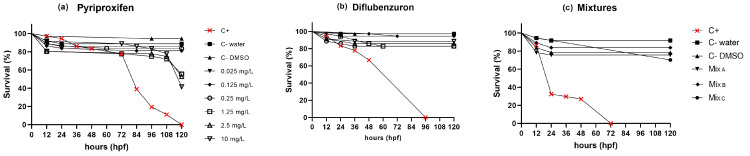
Survival rate of zebrafish embryos exposed to (**a**) Pyriproxyfen (PPF), (**b**) Diflubenzuron (DFB), and (**c**) PPF and DFB Mixtures for 120 h. Exposed animals were compared to control groups: Control water (C− water), Control DMSO 1% (C− DMSO), Control 3,4 Dichloroaniline (C+). Six concentrations of each pesticide were tested in isolation (0.025, 0.125, 0.25, 1.25, 2.5, and 10 mg/L). For the mixtures, PPF was combined with DFB to produce Mix A (0.025 mg/L PPF + 10 mg/L DFB), Mix B (0.125 mg/L PPF + 10 mg/L DFB), and Mix C (0.250 mg/L PPF + 10 mg/L DFB). Survival was monitored every 24 h (*n* = 36 embryos per group). The log-rank test revealed statistical significance for PPF (*p* < 0.05) but not for DFB and the mixtures.

**Figure 2:**
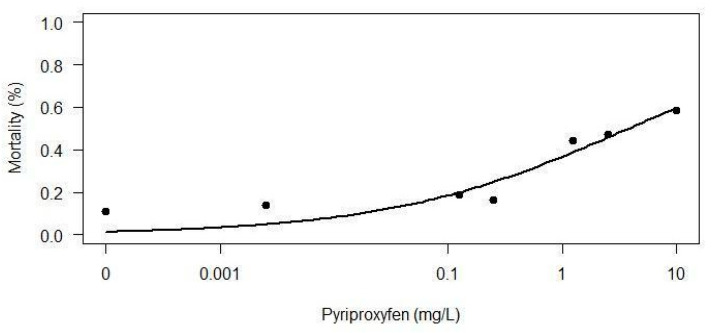
To determine the LC_50_ (Lethal Concentration 50) of pyriproxyfen, two exposures of Zebrafish embryos (*n* = 18 each) were conducted for up to 120 hpf, using the following concentrations: 0.025 mg/L, 0.125 mg/L, 0.25 mg/L, 1.25 mg/L, 2.5 mg/L, and 10 mg/L. LC_50_ calculated was 3.79 mg/L.

During 120 h, zebrafish embryos were exposed to pesticides or the control treatments. For the PPF assay, survival rate in the lower concentrations (0.025, 0.125 and 0.25 mg/L) did not differ from the control (*p* = 0.45), while survival of fish exposed to the highest concentrations, namely 1.25, 2.5, and 10 mg/L, was 55.5%, 52.7%, and 41.6%, respectively, all differed from the control (*p* = 0.0039; 0.0022; 0.003). The positive control showed 0% survival rate at 120 h. The negative control groups (water and DMSO) showed survival rates of 88.8% (water) and 94.4% (DMSO), respectively. The log-rank test showed statistical significance (*p* < 0.0001) (Figure 1a) and LC_50_ calculated is 3.79 mg/L for PPF (Figure 2). For DFB, there was no lethality during 120 h in any concentration, However, the positive control showed mortality of 100% (*p* < 0.0001) at 96 h (Figure 1b).

For the exposure to the mixtures, the positive control group showed a survival rate of 0% at 72 h, while the negative controls (water and DMSO) showed a survival rate of 91.6% and 78.3%, respectively. The log-rank test showed statistical significance (*p* < 0.0001) in the exposure groups for all treatments (Figure 1c) compared to positive control group. Comparing the concentrations used for the mixtures with their equivalents of PPF, the groups did not differ (*p* = 0.2586; *p* = 0.8036; *p* = 0.1583) from their respective concentrations of PPF (0.025, 0.125, and 0.25 mg/L).

### 3.2. Developmental Endpoints

**Figure 3:**
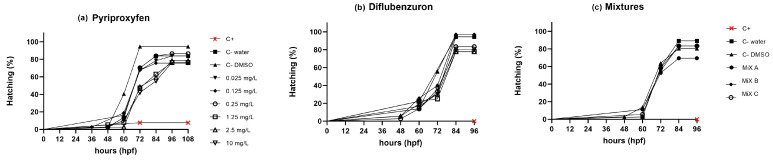
Hatching rate of zebrafish embryos exposed to (**a**) Pyriproxyfen (PPF), (**b**) Diflubenzuron (DFB), and (**c**) PPF and DFB Mixtures for 120 h. Exposed animals were compared to the control groups: Control water (C− water), Control DMSO 1% (C− DMSO), Control 3,4 Dichloroaniline (C+). Six concentrations of each pesticide were tested in isolation (0.025, 0.125, 0.25, 1.25, 2.5, and 10 mg/L). For the mixtures, PPF was combined with DFB to produce Mix A (0.025 mg/L PPF + 10 mg/L DFB), Mix B (0.125 mg/L PPF + 10 mg/L DFB), and Mix C (0.250 mg/L PPF + 10 mg/L DFB). The number of hatched eggs was monitored every 24 h (*n* = 36 embryos per group). The log-rank test revealed statistical significance for PPF, DFB, and the mixtures (*p* < 0.05).

Hatching rate data were statistically significant for DFB, PPF, and the mixtures (*p* < 0.0001). In the DFB assay, the negative controls (water and DMSO) started hatching at 60 hpf and reached their maximum level at 84 hpf (94.4% and 97.2%, respectively). The higher concentrations of DFB (1.25, 2.5, and 10 mg/L) exhibited the lower hatching rates at 96 hpf (77.7%, 77.7%, and 80.5%, respectively) (Figure 3a). For the PPF assay, the positive control presented the hatching peak at 72 hpf (7.6%), while the negative controls showed 80.2% (water) and 94.5% (DMSO) at the same time point. The highest hatching rate was observed for the concentration of 0.25 mg/L (86.4%), while the lowest rate was seen for 0.125 mg/L (75.6%) at 96 hpf (Figure 3b). For the mixtures assay, the hatching rate of the Mix A group was 69.4%, while Mix B and Mix C presented 83.3% hatching (both) at 96 hpf (Figure 3c). The positive control did not show any hatching, whereas the negative controls (water and DMSO) exhibited a hatching rate of 89.1% and 80.5% at 96 hpf, respectively.

**Figure 4:**
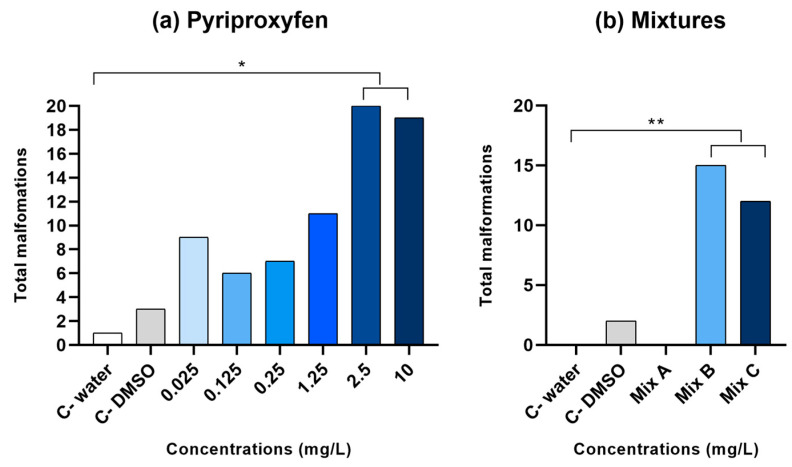
Total malformations of pyriproxyfen (**a**) and PPF + DFB Mixtures (**b**). Exposed animals were compared to the control groups: Control water (C− water) and Control DMSO 1% (C− DMSO). For the mixtures, PPF was combined with DFB to produce Mix A (0.025 mg/L PPF + 10 mg/L DFB), Mix B (0.125 mg/L PPF + 10 mg/L DFB), and Mix C (0.250 mg/L PPF + 10 mg/L DFB). The total number of malformations was observed throughout the 120 h (*n* = 36 embryos per group). One-way ANOVA was used to compare the mean malformations per group, revealing statistical significance (*p* < 0.05) indicated by the asterisks (* indicates *p* < 0.05; ** indicates *p* < 0.001).

Malformations were observed in larvae exposed to PPF (ANOVA, F (7, 239) = 3.117; *p* = 0.003). The post hoc test indicated a greater number of malformations in animals exposed to PPF at 1.25 and 2.5 mg/L (*p* < 0.05) (Figure 4a). Larvae treated with DFB did not show a number of malformations that were statistically significant compared to the water control group (*p* = 0.20). However, the groups exposed to the mixtures B (DFB 10 mg/L + PPF 0.125 mg/L) and C (DFB 10 mg/L + PPF 0.25 mg/L) presented a number of malformations that were statistically significant compared to the control group (ANOVA, F (4, 143) = 5.52; *p* < 0.0005) (Figure 4b).

Malformations identified throughout the exposure included sac and pericardial edema, eye, tail and bent malformations. Upon exposure to PPF, the main observed malformations were sac edema, pericardium, and head malformation at the concentrations of 1.25, 2.5, and 10 mg/L (two-way ANOVA, F (4, 28) = 4.14, *p* = 0.009) (Supplementary Data Figure S1). For DFB exposure, the number of malformations observed were not significant between groups (two-way ANOVA, F (4, 28) = 0.68, *p* = 0.61) (Supplementary Data Figure S1b). For the larvae treated with the mixtures of PPF and DFB, a higher occurrence of malformations was observed in the two higher concentrations of PPF (two-way ANOVA, F (4, 28) = 4.14, *p* = 0.008). Mix B (DBF 10 mg/L + PPF 0.125 mg/L) caused tail malformation and pericardial edema mainly, while Mix C (DBF 10 mg/L + PPF 0.25 mg/L) provoked higher number of sac edema and head malformations (Supplementary Data Figure S1c).

**Figure 5:**
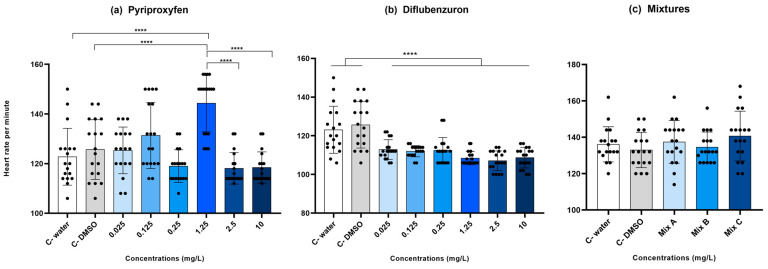
Cardiac function by heart rates in beats per minute (bpm) counted for embryos exposed to (**a**) Pyriproxyfen (PPF), (**b**) Diflubenzuron (DFB), and (**c**) PPF and DFB Mixtures for 120 h. Exposed animals were compared to the control groups; water (C− water) and DMSO 1% (C− DMSO). Six concentrations of each pesticide were tested in isolation (0.025, 0.125, 0.25, 1.25, 2.5, and 10 mg/L). For the mixtures, PPF was combined with DFB to produce Mix A (0.025 mg/L PPF + 10 mg/L DFB), Mix B (0.125 mg/L PPF + 10 mg/L DFB), and Mix C (0.250 mg/L PPF + 10 mg/L DFB). The number of larvae analyzed was 18 per group. **** indicates statistical significance between groups at *p* < 0.001.

For the cardiac function, heart rate per minute was obtained from 18 larvae per group. Fish treated with DFB presented a reduced heart rate compared to the control groups (ANOVA, F (7, 136) = 15.70, *p* < 0.0001), which rates were 123.1 and 125.6 beats per minute (bpm) for control water and control DMSO, respectively (Tukey test, *p* < 0.05) (Figure 5a). For the PPF exposure, the group treated with 1.25 mg/L presented an increased heart rate (144.3 bpm) compared to the control groups (122.7 and 125.6 bpm) and to the higher concentrations of PPF (ANOVA, F (7, 136) = 14.0, *p* < 0.0001) (Figure 5b). The mixtures of PPF and DFB did not cause a significant variation in heart rate (ANOVA, F (4, 85) = 1.33, *p* = 0.26) (Figure 5c). 

### 3.3. Behavioral Endpoints

**Figure 6:**
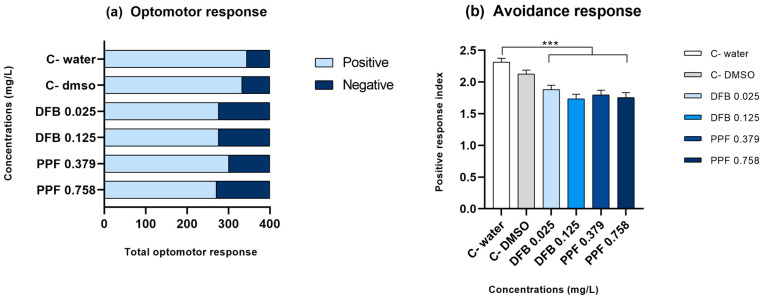
Zebrafish larvae behavioral responses related to (**a**) optomotor reflex and (**b**) avoidance behavior. Animals (*n* = 10 larvae per group) exposed to pesticides and the control groups (C− water and C− DMSO 1%) were tested 7 days after a 120 h exposure. For PPF exposure, concentrations of 0.379 and 0.758 mg/L, related to the LC_50_ (Lethal Concentration 50) calculated in acute exposure (3.79 mg/L), were used. For DFB, lower concentrations (0.025 and 0.125 mg/L) previously tested in the acute exposure experiment were used. For both tests, one-way ANOVA was used to compare the mean malformations per group. The optomotor response test did not show statistical significance (*p* = 0.62), while the avoidance response test revealed statistical significance, as indicated by *** (*p* < 0.001).

The optomotor response of larvae was recorded and compared among treatments at 7 days post-fertilization (dpf). One-Way ANOVA did not reveal a statistically significant difference between groups (F (5, 6) = 0.73, *p* = 0.62; Figure 6a). Figure 6b depicts the avoidance response of larvae treated with the pesticides PPF and DFB. One-Way ANOVA showed statistical significance between groups (F (5, 1194) = 11.30, *p* < 0.0001). The post hoc test indicated that the control groups (water and DMSO) presented heightened avoidance behavior in comparison to the fish exposed to the pesticides (*p* < 0.05). This implies that the control fish moved farther away from the stimulus than the treated fish.

### 3.4. ROS

**Figure 7:**
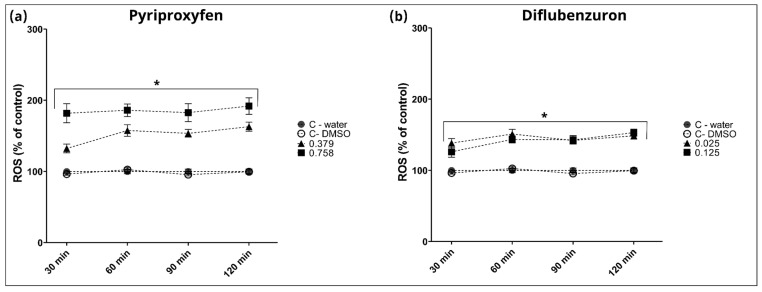
ROS levels after 120 hpf using H_2_DCF-DA. (**a**) pyriproxyfen (PPF) at concentrations of 0.379 and 0.758 mg/L, and (**b**) diflubenzuron (DBF) at concentrations of 0.025 and 0.125 mg/L. ROS concentration was expressed as a percentage (%) of fold change compared to control at 30, 60, 90, and 120 min. Results are represented as mean ± SEM; asterisk indicates statistical significance (*p* < 0.05).

Two-way ANOVA revealed a significant increase in reactive oxygen species (ROS) levels in larvae exposed to the pesticides PPF and DFB, as shown in Figure 7. Exposure to PFF resulted in significant changes in ROS production at all observed time points compared to the negative control and DMSO, as depicted in Figure 7a, with a significant interaction between groups and time [F (9, 105) = 7.99, *p* < 0.0001], confirmed by a Dunnett’s post hoc test. Similarly, exposure to DFB also induced significant alterations in ROS production compared to the negative controls, with a significant interaction between groups and time [F (9, 105) = 7.05, *p* < 0.0001], supported by a Dunnett’s post hoc test, as shown in Figure 7b.

## 4. Discussion

This study aimed to conduct a thorough analysis of the embryotoxicity of two pesticides widely used in crops, as well as their mixtures, utilizing the zebrafish embryo as a model. The investigation involved subjecting embryos to various concentrations of the pesticides PPF and DFB individually and in combination for a duration of 120 h. Zebrafish larvae were employed to assess the impact of these agrochemicals, complemented by neurotoxicity tests and an evaluation of oxidative stress. The outcomes of this study contribute to an understanding of the safety profile of these pesticides and their potential impacts on both human and animal health.

We determined the LC_50_ for PPF to be 3.79 mg/L, while the LC_50_ for DFB was not ascertainable. The highest concentration of DFB (10 mg/L), which was soluble in 1% DMSO, did not induce embryo mortality. Concentrations beyond this limit were insoluble in DMSO, even with increased concentrations or usage of any other solvent (methanol, ethanol, acetone and hexane). This issue limited our ability to dissolve the chemical and determine LC_50_. Different from our methodology, Dantzger et al. [24] used the commercial product in its standard formulation (Dimilin^®^, Chemtura Industria Quimica Ltd.a, São Paulo, Brazil) when conducting their study. These authors combined DFB with p-chloroaniline, capitalizing on the industrial solubilization, a process not explicitly outlined in the commercial product’s leaflet. In their study, the concentrations used for both chemicals were: 0.1, 1.0, 10.0, and 100.0 mg/L; however, toxic effects were not observed during the exposure to the commercial formulation of DFB. On the contrary, Han et al. [25] employed dimethyl sulfoxide (DMSO) to dissolve DFB at concentrations of 0.5, 1.5, and 2.5 mg/L, demonstrating its solubility power at lower concentrations, corroborating our methods.

For the assessment of survival rates, we noted that PPF concentrations below 0.25 mg/L exhibited no discernible effects, comparable to the control groups. However, concentrations surpassing 1.25 mg/L led to a reduction in embryo survival. Notably, no lethality was observed at any DFB concentration. Consequently, we conducted tests on the survival of embryos exposed to mixtures, combining the higher concentration of DFB (10 mg/L) with PPF concentrations that did not induce mortality (0.025, 0.125, and 0.25 mg/L). Surprisingly, no effects were observed for the mixtures, suggesting that, regarding survival outcomes, the pesticides do not interact to provoke effects that threaten survival. However, by employing the soluble concentrations of PPF and DFB, we noted that hatching time was significantly affected at values below the LC_50_ (0.125 mg/L) for PPF and concentrations higher than 1.25 mg/L for DFB. When incorporating mixtures, even the lowest mixture resulted in a hatching delay. 

The safety assessment of PPF and DFB was conducted during a critical developmental phase, namely gastrulation. Both survival and hatch rates were considered crucial, as reductions in these parameters might signify potential toxicity [26]. Moreover, the examination revealed numerous morphological defects in larvae exposed to these chemicals, constituting a significant finding. Teratogenicity emerges as a primary concern in evaluating the biosafety of compounds during embryonic development [27]. Our study suggests that PPF concentrations surpassing 2.5 mg/L induce teratogenic effects, and a concentration as low as 0.125 mg/L of PPF, when mixed with DFB, is sufficient to cause body malformations when exposed during the initial 96 h of zebrafish embryo development. The increased number of malformations observed for the mixtures suggest the PPF and DFB together present potentiating effects. Similarly, in a study by Kannan et al. [28], zebrafish embryos were exposed to 0.16, 0.33, and 1.66 μg/mL of PPF for 96 h and the findings revealed severe developmental deformities and alterations in heart rate in embryos treated with 1.66 μg/mL. In addition to this, Maduenho and Martinez [10] tested the effects of 25 mg/L of DFB (commercial formulation, Dimilin^®^) in adult curimbatá fish, *Prochilodus lineatus*, demonstrating a reduction in the number of erythrocytes and hemoglobin content after 96 h of exposure.

In our study, the cardiac function of the larvae was assessed as complementary data to the teratogenic results, given that the heart is among the first organs to develop in zebrafish, influencing the functioning of various other tissues [18]. Regarding the heart, only the highest concentration of DFB caused impairment, potentially leading to mortality with prolonged exposure. Both the mixtures and most isolated PPF concentrations did not induce alterations in heart rate, but PPF at 1.25 mg/L resulted in an increase in heart beating in zebrafish. This outcome may suggest the presence of the hormesis phenomenon, wherein exposure to a low concentration is harmful, and elevated concentrations trigger various other organic responses (such as cortisol release and increased metabolic demands) that counteract the initial effect. Similar hormesis effects have been observed with other toxins and drugs, exemplified by oxybenzone [29]. These authors showed that adult zebrafish exposed to oxybenzone at 10µg/L presented increased locomotor activity, while higher concentrations did not cause hyperactivity, suggesting an interaction of the drug with other organic systems, as the endocrine system. Endocrine disruptors such as glyphosate [30], silver nitrate [31], and tebuconazole [32] were shown to induce the same inverted U curve response as observed here for PPF.

In addition to all the morphological implications of the pesticides exposure, several effects of toxics are not visible in a macro scale. In this sense, neurotoxicity tests and behavioral expression are interconnected components when assessing the impact of toxics on the nervous system. The relationship between these two lies in the fact that behavioral changes can be indicative of neurotoxic effects as changes in behavior precede detectable physiological or structural damage to the nervous system. Moreira et al. [33] demonstrated alterations in learning and memory after exposure to oxybenzone (BP-3) in zebrafish for 15 days. Neurotoxicity can manifest as alterations in cognitive function, motor coordination, or other behavioral patterns. In this study, we assessed neurotoxicity by the optomotor and avoidance responses. 

While the cognitive repertoire of zebrafish larvae is more limited compared to adult fish, they exhibit specific vision-oriented cognitive responses with well-validated protocols for assessing reflex responses and cognitive processing [20,33,34,35,36]. The optomotor response, a well-described reflex orientating behavior, involves zebrafish larvae moving towards visual motion patterns [37,38].

In this study, we observed that although the optomotor response, which refers to a characteristic swimming movement in response to visual stimuli, indicates the animal sensory-motor integration it is not affected by the pesticides. However, when presented with a threatening stimulus that requires perception and avoidance [39] the animals did not perform as the control group. This result shows that zebrafish larvae exposed to PPF and DFB had a diminished capacity to avoid hazardous situations. This observed reduction in the ability to swim away from aversive stimuli supports the notion of a negative impact of pesticides on the larvae’s nervous system and the ability to perceive and avoid exposure to risk.

Despite its relative simplicity, the avoidance response can serve as a reliable tool for behavioral analysis of cognitive and learning processes in zebrafish larvae [20,34]. The results obtained in this study strongly indicate damage caused by pesticides to the nervous system. Molecular endpoint studies can be crucial in elucidating the underlying pathways, as changes at the molecular level cannot be excluded.

In this study, an increase in ROS generation was observed with the use of PPF and DFB, as evidenced by the H_2_DCF–DA approach. Although H_2_DCF–DA is a non-specific ROS indicator, the results shown in Figure 7 highlights a significant difference between the control group and the treatments exposed to both agrochemicals. In a study involving adult zebrafish, PPF also increased the generation of ROS [40]. Researchers adopted two distinct approaches, using H_2_DCF–DA and MitoSOX, to investigate the impact of PPF on ROS production. When employing H_2_DCF–DA, a clear difference was shown between the control group and groups exposed to PPF. In the other approach, using MitoSOX to detect mitochondrial superoxide anion, it was observed that PPF exerts a dose-dependent effect on generating this mitochondrial superoxide anion. In another in vitro study, DFB induced apoptotic cell death through the generation of ROS and mitochondrial dysfunction in bovine mammary epithelial (MAC-T) cells. Furthermore, DFB-induced ROS affected the loss of mitochondrial membrane potential (MMP), calcium ion homeostasis, and PI3K/AKT and MAPK signaling. Collectively, these factors contributed to decreased cell viability and cell death in MAC-T cells exposed to DFB [41].

Overall, our results highlight the adverse impacts of PPF and DFB, both individually and in their mixtures, on the development and functioning of non-target animals, as demonstrated using the zebrafish model. One limitation observed in this study was the inability to further explore tests with DFB due to solubility issues, which hindered increasing the concentrations. Solubilization studies need to be conducted and evaluated in water, along with tests of bioaccumulation or bioconcentration in the tissues of the animal model, which we were unable to perform. In rats, it was demonstrated that diflubenzuron can accumulate in the liver and adipose tissue, and rarely in other tissues, due to its high rate of metabolism, presenting a low bioaccumulation potential [42]. This was also demonstrated by Ref. [43] that in experiments with Eremias argus, showed bioaccumulation in adipose tissue and brain, but with a high rate of metabolism in tissues. Regarding Piriproxyfen [44], they showed that zebrafish are quite resistant to pyriproxyfen, and despite bioaccumulating in tissues, it is also metabolized, and its secondary metabolites are harmful to tissues. The solubility of these substances poses a challenge when assessing their effects, and attention must be given to the effects of particles. Additionally, we were unable to provide results on the effects of the mixtures on neurotoxicity (behavior and ROS), thereby limiting the understanding of the adverse effects of pesticide combinations. Nevertheless, various other pesticides are simultaneously employed in crops to manage different pests or address distinct ontogenetic phases of the pests. Additionally, commercial products containing active compounds, such as PPF and DFB, may encompass diverse chemical substances that interact with both the active compound and the target (or non-target) organisms. Consequently, future studies should delve into the examination of other mixtures, variations in commercial and active compounds, exploration of diverse developmental phases of non-target animals, and considerations of long-term effects and specific tissue impacts. Such comprehensive analyses are crucial for evaluating the viability of pesticides.

## 5. Conclusions

The analysis of the effects of the agrochemicals DFB and PPF, both individually and in mixtures, along with behavioral assessments, highlights the complexity of interactions between chemicals and organisms with suggested potentiating effects. Our data have demonstrated the potential toxicity of these substances on the embryonic development of zebrafish embryos, providing neurotoxic results related to the behavior and redox imbalance. The detrimental effects of PPF and DFB exposure suggest the necessity to reconsider these pesticides consumption. Exploring less impactful substances for pest control warrants careful attention and thorough testing as potential alternatives to the aforementioned pesticides. These substances should ensure the safety and protection of both animals and the environment.

## Data Availability

All data will be made available upon request.

## Supplementary Materials

The following supporting information can be downloaded at: https://www.mdpi.com/article/10.3390/toxics12020160/s1, Figure S1: Detailed total malformations of pyriproxyfen, diflubezuron and its mixtures.
