# Supplementary material for: Embryotoxic Effects of Pesticides in Zebrafish (Danio rerio): Diflubenzuron, Pyriproxyfen, and Its Mixtures
Source: Toxics. 2024 Feb 18;12(2):160. doi: 10.3390/toxics12020160 (PMC10892354; doi:10.3390/toxics12020160)
Supplement: Supplementary file 1 [file toxics-12-00160-s001.zip › toxics-2863305-supplementary.pdf]

Supplementary Data

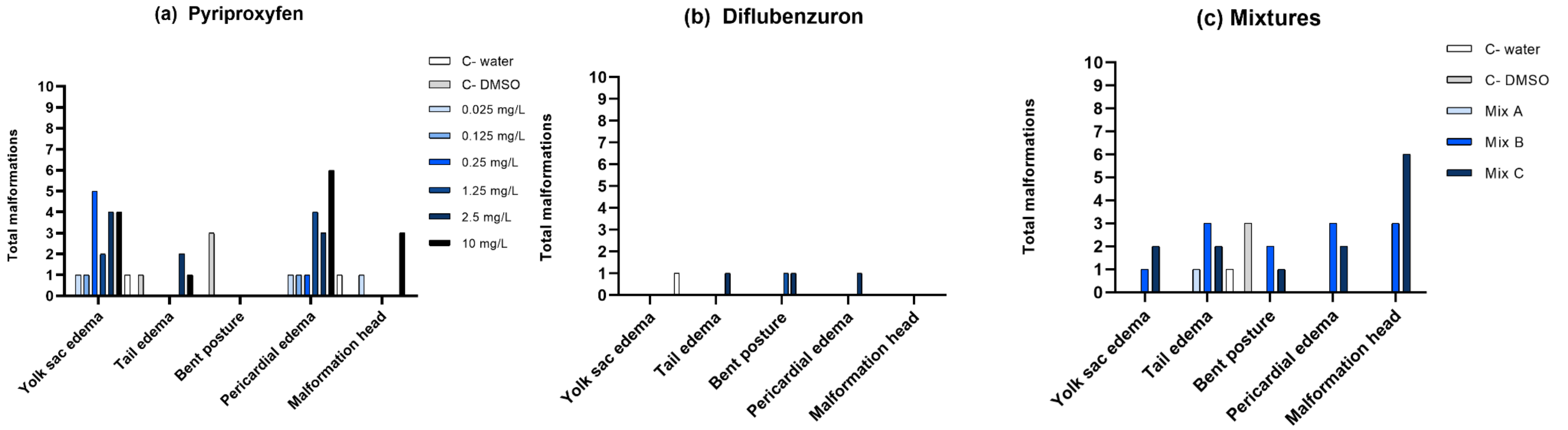

Figure S1. Detailed total malformations of pyriproxyfen (a), diflubenzuron (b), and its mixtures (c). Exposed animals were compared to control groups: Control water (C-water) and Control DMSO 1%. For the mixtures, PPF was combined with DFB to produce Mix A (0.025mg/L PPF + 10 mg/L DFB), Mix B (0.125mg/L PPF + 10 mg/L DFB), and Mix C (0.250mg/L PPF + 10 mg/L DFB). The animals were observed for 120 hours (n=36 animals per group), revealing the types of malformations present in the group, including yolk sac and pericardial edema, as well as head, spine, and tail malformations. Two-way ANOVA was conducted to compare the groups, indicating significance for all treatments ($p < 0.05$).
